# Randomized clinical trial of artemisinin versus non-artemisinin combination therapy for uncomplicated falciparum malaria in Madagascar

**DOI:** 10.1186/1475-2875-6-65

**Published:** 2007-05-22

**Authors:** Didier Ménard, Nohary Nina Harimanana Andrianina, Zakaherizo Ramiandrasoa, Arthur Randriamanantena, Noéline Rasoarilalao, Martial Jahevitra, Arsène Ratsimbasoa, Luciano Tuseo, Andrianirina Raveloson

**Affiliations:** 1 Malaria Unit Research, Institut Pasteur de Madagascar, BP 1274, Antananarivo 101, Madagascar; 2 Epidemiology Unit, Institut Pasteur de Madagascar, Antananarivo 101, Madagascar; 3 Roll Back Malaria, WHO Office of Madagascar and La Réunion, Antananarivo 101, Madagascar; 4 National Malaria Control Programme, Ministry of Health, Antananarivo 101, Madagascar

## Abstract

**Background:**

Data concerning antimalarial combination treatment for uncomplicated malaria in Madagascar are largely lacking. Randomized clinical trial was designed to assess therapeutic efficacies of chloroquine (CQ), amodiaquine (AQ), sulphadoxine-pyrimethamine (SP), amodiaquine plus sulphadoxine-pyrimethamine combination (AQ+SP) and artesunate plus amodiaquine combination (AQ+AS).

**Methods:**

287 children between 6 months and 15 years of age, with uncomplicated falciparum malaria, were enrolled in the study. Primary endpoints were the day-14 and day-28 risks of parasitological failure, either unadjusted or adjusted by genotyping.

**Results:**

All treatment regimens, except for CQ treatment, gave clinical cure rates above 97% by day-14 and 92% by day-28 (PCR-corrected). AQ+SP was as effective as AQ+AS. The risk of new infection within the month after therapy was generally higher for AQ+AS than AQ+SP.

**Conclusion:**

These findings show that the inexpensive and widely available combination AQ+SP may be valuable in for the treatment of uncomplicated malaria in Madagascar and could have an important role in this country, where much of the drugs administered go to patients who do not have malaria.

## Background

Malaria remains one of the most serious health problems worldwide and a leading cause of childhood morbidity and mortality, especially in Africa [1]. At least 300 million clinical cases of malaria occur each year, resulting in more than a million child deaths worldwide. Early diagnosis and prompt effective treatment remain the cornerstone for the reduction of malaria-related morbidity and mortality [2]. However, efforts to control malaria in Africa have been severely compromised by the emergence of resistance in *Plasmodium falciparum *to the inexpensive and widely used drugs, chloroquine (CQ) and sulphadoxine-pyrimethamine (SP) [3,4]. As a consequence, the use of combination antimalarial therapy has been widely advocated [5]. Of the available antimalarial drugs, the artemisinins are the most potent and the World Health Organization (WHO) specifically advocates the use of artemisinin-based combination therapy (ACT) as the standard policy for the treatment of uncomplicated falciparum malaria [6]. ACTs give a rapid clinical and parasitological response, may delay the development of resistance and may reduce malaria transmission by killing gametocytes. However, there are concerns regarding the cost and availability of artemisinin-based combination therapy (ACT), and only limited data comparing ACT with other combination therapies in Africa are available [7]. Moreover, it is unclear whether ACTs are appropriate for empirical management of febrile illnesses outside the formal health sector without laboratory confirmation, in the way that CQ and SP have been used for several years.

Malaria treatment in Madagascar is in transition: for over 50 years, malaria was treated successfully using chloroquine (CQ) as first-line treatment. In 2005, the National Malaria Control Programme (NMCP) decided to revise its treatment policy and to replace CQ with artemisinin-based combination therapy (AQ+AS, artesunate plus amodiaquine combination) with the support of resources from the Global Fund. This choice was guided by the recommendations of WHO and data from the most recent clinical trial based on the WHO standard protocol [8]: the trial was conducted on the island of Sainte Marie in 2004 and has demonstrated clinical failure in 36.9% of cases within two weeks of CQ treatment. Alternatives to ACTs, which include the non-artemisinin-based combination therapy (NACT) amodiaquine plus sulphadoxine/pyrimethamine (AQ+SP), were surprisingly not considered, although the WHO's Roll Back Malaria programme had recommended the use of NACT in settings where the efficacy of both component drugs is high; this was the case in Madagascar [9,10].

Few data is available concerning alternative or combinatory antimalarial treatment in uncomplicated malaria in Madagascar. Randomized clinical trial was designed (i) to update knowledge about the therapeutic efficacies of CQ (used for the home management of presumed malaria in children under the age of five years), AQ and SP alone (used for intermittent preventive treatment in pregnant women), (ii) to compare the respective therapeutic efficacies of two combinatory treatments, AQ+SP and AQ+AS, and (iii) to generate data which could be used as a guide for designing a rational antimalarial treatment policy in Madagascar.

## Materials and methods

### Study design and study sites

The study was conducted between February and June 2006 during and at the end of the rainy season, in primary health centres of Moramanga and Saharevo in the east foothill areas of the Highlands of Madagascar. In this area, malaria transmission is low and predominantly seasonal. The main vector is *Anopheles funestus *and the number of infective bites associated with *Plasmodium falciparum *is estimated at < 10 per person per year. The study protocol was reviewed and approved by the Ethics Committee of the Ministry of Health of Madagascar (N°007/SANPF/2007).

### Patients

Children between six months and 15 years of age presenting at primary health centres of Moramanga and Saharevo were enrolled in the study if they met the following inclusion criteria [11]: (i) monoinfection with *P. falciparum *at a parasitaemia between 1,000 and 200,000/μl, (ii) axillary temperature ≥ 37.5°C, (iii) body weight > 5 kg, (iv) absence of severe malnutrition, (v) absence of febrile conditions caused by diseases other than malaria, (vi) absence of 'danger signs' (inability to stand, breastfeed or drink; recent convulsions; lethargy or persistent vomiting) and of severe and complicated malaria, (vii) haemoglobin (Hb) ≥ 5 g/dl, and (viii) informed written consent of parents/guardians.

Exclusion criteria were: (i) known hypersensitivity to SP, AQ, or AS, (ii) detection, during follow-up of mixed malarial infections, and (iii) development of concomitant disease which would interfere with the classification of treatment outcome.

### Treatments, randomization and blinding

Patients were randomly assigned to receive one of five oral therapies: CQ (10 mg/kg on days 0 and 1, and 5 mg/kg on day 2); AQ (10 mg/kg on days 0, 1, and 2); SP (25 mg/kg sulphadoxine and 1.25 mg/kg pyrimethamine as a single dose on day 0); AQ + SP or AQ + AS (4 mg/kg on days 0, 1, and 2). Randomization was performed in blocks of five, and treatment regimens were allocated by an independent individual not involved in the analysis of the study.

All other study personnel were blinded to the treatment assignments, and patients were not informed of their treatment regimen. Patients were directly observed for 30 minutes after treatment, and the dose was readministered if vomiting occurred. Patients who repeatedly vomited their first dose of study medication were excluded from the study.

### Follow-up procedures and classification of treatment outcomes

Following enrollment, patients were asked to return for follow-up visits on days 1, 2, 3, 7, 14, 21, 28, and any other day if they felt ill. Blood was obtained by finger prick for thick blood smears and storage on filter paper on all follow-up days. Haemoglobin was determined on day 0 and on day 28. Treatment outcomes were classified according to 2003 WHO guidelines as Early Treatment Failure (ETF; danger signs or complicated malaria or failure to adequately respond to therapy on days 0–3), Late Clinical Failure (LCF; danger signs or complicated malaria or fever and parasitaemia on days 4–28 without previously meeting criteria for ETF), Late Parasitological Failure (LPF; asymptomatic parasitaemia on days 4–28 without previously meeting criteria for ETF or LCF), and Adequate Clinical and Parasitological Response (ACPR; absence of parasitaemia on day 28 without previously meeting criteria for ETF, LCF, or LPF) [11].

Patients classified as having suffered treatment failure were treated with quinine (10 mg/kg three times daily for 7 days); however, their response to repeat therapy was not assessed. Patients were excluded after enrollment if any of the following occurred: (1) use of antimalarial drugs outside of the study protocol; (2) parasitaemia in the presence of a concomitant febrile illness; (3) withdrawal of consent; (4) loss to follow-up, (5) protocol violation, or (6) death due to a non-malaria illness.

### Laboratory procedures

Blood smears were stained with 4 % Giemsa for 20 min. Parasite densities were determined from thick blood smears by counting the number of asexual parasites per 200 WBCs (or per 500, if the count was less than 10 parasites/200 WBCs), assuming a WBC count of 8,000/μl. A smear was considered negative if no parasites were seen after review of 100 fields. Thin blood smears were used to detect non-falciparum infections. Gametocytes were counted against 500 WBC for 287 children at enrolment and in 258, 257, 220, 217 specimens available for that purpose collected on day 7, day 14, day 21 and day 28, respectively. A portable spectrophotometer (HemoCue^©^, Anglholm, Sweden) was used for haemoglobin assays.

Molecular genotyping techniques were used to distinguish recrudescence from new infection for all patients failing therapy after day 7. Briefly, filter paper blood samples collected on the day of enrollment, on day 1 and on the day of failure were analyzed for polymorphisms in the genes for merozoite surface protein-1 (MSP-1) and merozoite surface protein-2 (MSP-2) using nested-PCR as previously described [12]. First, MSP-2 genotyping patterns on the day of failure were compared with those at treatment initiation and on day 1, using Quantity One^© ^software (BioRad laboratories, Inc., 1000 Alfred Nobel Drive, Hercules, CA 94547, United States). If all of the MSP-2 alleles present on the day of failure were present at the time of treatment initiation or on day 1, genotyping was repeated using MSP-1. An outcome was defined as recrudescence if all MSP-1 and MSP-2 alleles present at the time of failure were present at the time of treatment initiation or on day 1, and defined as a new infection otherwise.

### Statistical analysis

Data were entered and verified using EpiInfo 6.04^© ^software (Centers for Disease Control and Prevention, Atlanta, Georgia, United States), and analysed using MedCalc^© ^software version 9.1.0.1 (MedCalc Software, Broekstraat 52, 9030 Mariakerke, Belgium).

Analysis of treatment outcome was per protocol, which only included patients with treatment outcomes. Frequencies were compared by chi-squared tests and Fisher exact tests, and continuous variables by Student's t-tests, Mann-Whitney U-tests, analysis of variance or Kruskal-Wallis tests as applicable. All reported p-values are two-sided, without adjustment for multiple testing, and were considered statistically significant if less than 0.05.

## Results

**Figure 1:**
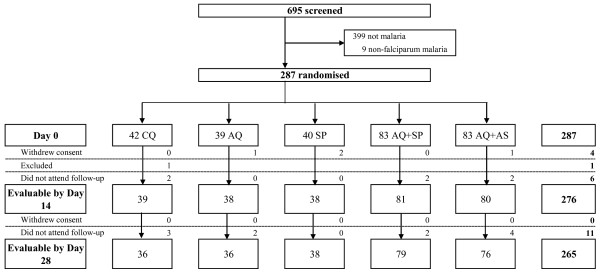
Trial profile.

695 patients were screened for inclusion in the study and 287 patients were randomized to receive CQ, AQ, SP, AQ + SP or AT + AQ (Figure 1). The baseline characteristics of the patients assigned to the five treatment regimes were similar (Table 1).

**Table 1:** Baseline characteristics of patients

	**Treatment group**				
	
**Parameters**	**CQ**	**AQ**	**SP**	**AQ+SP**	**AQ+AS**
No. of patients	42	39	40	83	83
No. females (%)	20 (47.6%)	22 (56.4%)	26 (65.0%)	42 (50.6%)	38 (46.3%)
Mean age (months. range)	3.8 (0.6–14)	3.3 (0.7–13)	3.8 (0.5–12)	3.9 (0.8–15)	3.9 (0.5–15)
Mean weight (kg. range)	12.9 (7–35)	13.0 (7.8–40)	12.1 (6–34)	14.3 (7–50)	14.6 (6–54.5)
Mean temperature (°C. range)	38.4 (37.5–40.4)	38.4 (37.5–40.8)	38.5 (37.5–40.8)	38.6 (37.5–41.0)	38.6 (37.5–40.9)
Geometric mean parasite density (/μl. range)	21156 (1020–199058)	25559 (1142–199800)	25640 (1010–199531)	22368 (1050–199048)	22905 (1005–184881)
Previous antimalrial therapy (%)	2 (4.7%)	1 (2.9%)	2 (5.0%)	2 (3.9%)	3 (5.3%)
Mean haemoglobin (g/dl. range)	10.1 (6.2–14.0)	10.0 (6.4–14.2)	9.7 (6.2–12.4)	10.2 (6.2–15.6)	10.4 (6.2–15.5)

CQ. Chloroquine; AQ. Amodiaquine; SP. Sulphadoxine-pyrimethamine; AS. artesunate.

Clinical and parasitological monitoring was complete for 96.1% (276/287) until day 14 of follow-up and for 92.3% (265/287) until day 28. Seventeen patients (5.7%) were lost to follow-up, four patients withdrew consent (1.3%) and one patient (0.3%) had to be withdrawn because he was treated outside the study with drugs active against malaria (Figure 1).

No severe side-effects attributable to the study medication were observed during the follow-up period, except that one patient treated with AQ+SP developed vomiting.

The efficacy of test drugs against uncomplicated falciparum malaria is shown in Table 2. All treatment regimens, except for the CQ treatment group, resulted in clinical cure rates above 97% by day-14 and 92% by day-28 (PCR-corrected). ETF was significantly less common in the two combinatory treatment (AQ+SP and AQ+AS) groups than in the CQ group on day 14 and day 28 (PCR-corrected and -uncorrected); LCF was significantly less common in AQ, SP, AQ+SP and AQ+AS treatment groups on day 14 and in SP, AQ+SP and AQ+AS on day 28 (PCR-uncorrected) and in only the two combinatory treatments on day-28 (PCR-corrected); ACPR to AQ, SP, AQ+SP and AQ+AS did not differ significantly on day-14 or day-28 (PCR-uncorrected and -corrected).

**Table 2:** Classification of treatment outcome

		**Treatment group**									
**Treatment outcome**		**CQ**		**AQ**		**SP**		**AQ+SP**		**AQ+AS**	
**14-day follow-up**											
ETF (%)		4/39 (10.3)		0/38 (0)		0/38 (0)		0/81 (0)*		0/80 (0)*	
LCF (%)	OTF (%)	7/39 (17.9)	**14/39 (35.9)**	0/38 (0)*	**0/38 (0)**	0/38 (0)*	**1/38 (2.6)**	0/81 (0)*	**1/81 (1.2)**	0/80 (0)*	**0/80 (0)**
LPF (%)		3/39 (7.7)		0/38 (0)		1/38 (2.6)		1/81 (1.2)		0/80 (0)	
ACPR (%)		**25/39 (64.1)**		38/38 (100)*		37/38 (97.4)*		80/81 (98.8)*		80/80 (100)*	
**28-day follow-up non adjusted (PCR-uncorrected)**											
ETF (%)		4/36 (11.1)		0/36 (0)		0/38 (0)		0/79 (0)*		0/76 (0)*	
LCF (%)	OTF (%)	9/36 (25.0)	**20/36 (55.5)**	3/36 (8.3)	**5/36 (13.9)**	0/38 (0)*	**2/38 (5.2)**	2/79 (2.5)*	**4/79 (5.0)**	3/76 (3.9)*	**12/76 (15.7)**
LPF (%)		7/36 (19.4)		2/36 (5.6)		2/38 (5.2)		2/79 (2.5)*		9/76 (11.8)	
ACPR (%)		**16/36 (44.5)**		31/36 (86.1)*		36/38 (94.8)*		75/79 (95.0)*		64/76 (84.3)*	
**28-day follow-up adjusted (PCR-corrected)**											
New infections (%)		4/36 (11.1)		4/36 (11.1)		1/38 (2.6)		1/79 (1.2)**		6/76 (7.9)	
ETF (%)		4/36 (11.1)		0/36 (0)		0/38 (0)		0/79 (0)*		0/76 (0)*	
LCF (%)	OTF (%)	7/36 (19.4)	**13/36 (44.4)**	1/36 (2.8)	**1/36 (2.8)**	0/38 (0)	**1/38 (2.6)**	1/79 (1.3)*	**3/78 (3.8)**	1/76 (1.3)*	**6/76 (7.9)**
LPF (%)		5/36 (13.9)		0/36 (0)*		1/38 (2.6)		2/79 (2.5)*		5/76 (6.6)	
ACPR (%)		**20/36 (55.6)**		35/36 (97.2)*		37/38 (97.4)*		76/79 (96.2)*		70/76 (92.1)*	

Data presented are cumulative, failures up to day 14 of follow-up are also considered in the calculation of day 28 failure rates.

Percentages are rounded to achieve totals of 100%.

CQ, Chloroquine; AQ, amodiaquine; SP, Sulphadoxine-pyrimethamine; AS, artesunate ETF, early treatment failure; LCF, late clinical failure; LPF, late parasitological failure; ACPR, adequate clinical and parasitological response.

* Significant difference to CQ group (P < 0.05); ** Significant difference to CQ and AQ groups (P < 0.05).

The proportion of re-infections among recurring parasitaemia was higher with CQ (11.1%; 4/36) and AQ (11.1%; 4/36) than with AQ+SP (1.2%; 1/79; P = 0.04) (Table 2).

**Figure 2:**
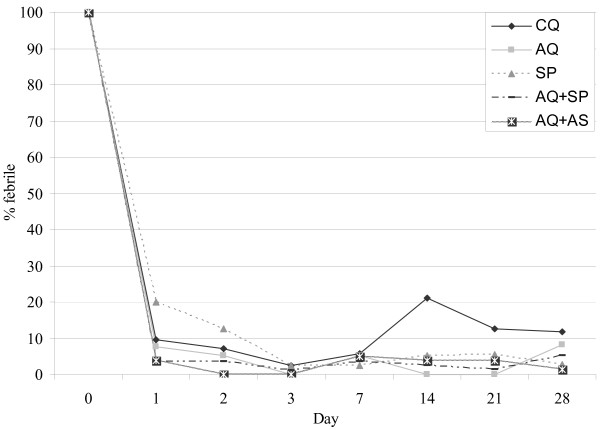
**Proportions of febrile patients following treatment**. CQ, Chloroquine, AQ, Amodiaquine; SP, Sulfadoxine-Pyrimethamine; AS, artesunate; On day 1, Significant difference to SP group (P < 0.05), SP vs. AQ+SP, SP vs. AQ+AS; At day 2, Significant difference to SP group (P < 0.05), SP vs. AQ+AS; On day 14, Significant difference to CQ group (P < 0.05), CQ vs. AQ, CQ vs. SP, CQ vs. AQ+SP, CQ vs. AQ+AS

Parasite clearance was more effective with AQ+AS than CQ, AQ or AQ+SP and with SP than CQ until day 2, but less effective with AQ+AS than AQ+SP on day 28 (Figure 2).

**Figure 3:**
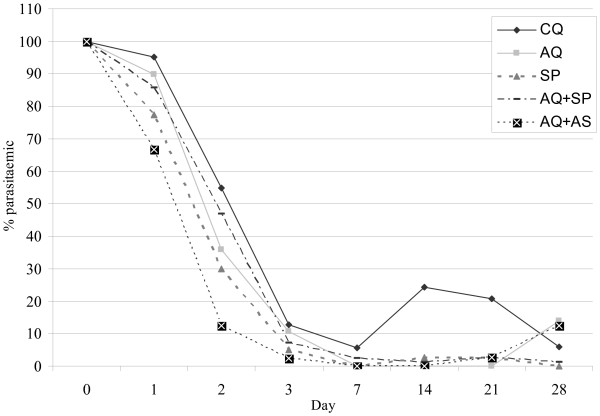
**Proportions of parasitaemic patients following treatment**. CQ, Chloroquine, AQ, Amodiaquine; SP, Sulfadoxine-Pyrimethamine; AS, artesunate; On day 1, Significant difference to CQ group (P < 0.05), CQ vs. SP, CQ vs. AQ+AS; Significant difference to AQ group (P < 0.05), AQ vs. AQ+AS; Significant difference to AQ+SP group (P < 0.05), AQ+SP vs. AQ+AS; On day 2, Significant difference to SP group (P < 0.05), SP vs. CQ; Significant difference to AQ+AS group (P < 0.05), AQ+AS vs. CQ, AQ+AS vs. AQ, AQ+AS vs. SP, AQ+AS vs. AQ+SP; on day 14, Significant difference to CQ group (P < 0.05), CQ vs. AQ, CQ vs. SP, CQ vs. AQ+SP, CQ vs. AQ+AS; On day 21, Significant difference to CQ group (P < 0.05), CQ vs. AQ, CQ vs. AQ+SP, CQ vs. AQ+AS; On day 28, Significant difference to AQ+SP group (P < 0.05), AQ+SP vs. AQ+AS

Fever clearance was delayed with SP alone, the proportion of febrile children being significantly lower with AQ+AS and AQ+SP until day-2 and day-1, respectively (Figures 2 and 3).

On day-28, the extent of haematological recovery (median of individual increases in Hb) did not differ significantly between the five groups (CQ, 0.8 g/dl, -0.8 to 3.4; SP, 1.2 g/dl, -1.0 to 5.8; AQ, 0,9 g/dl, -3.6 to 4.1; AQ+SP, 0.5 g/dl, -4.4 to 5.8; AQ+AS, 1.1 g/dl, -2.6 to 5.2).

Gametocyte prevalence at enrolment was 5.6% (16/287) with a geometric mean density of 124.7 gametocytes/μl (95% confidence interval, 50.5–307.9). There were no significant differences at enrolment in gametocyte prevalence or density with respect to treatment regime (Table 3). Treatment with SP gave significantly higher gametocyte prevalence on day-7 and on day-14 than the other treatments. On day-21, gametocyte prevalence in SP group was significantly higher than the two combinatory treatments (AQ+SP and AQ+AS).

**Table 3:** Gametocyte prevalence on days 0, 7, 14, 21 and 28 after treatment in patients with adequate parasitological response

	**Gametocyte prevalence, %, (n/N)**				
	
		**After treatment**			
		
	**At enrolment**	**Day 7**	**Day 14**	**Day 21**	**Day 28**
**CQ**	4.7 (2/42)	12.5 (3/24)*	4.2 (1/24)*	6.6 (1/15)	0 (0/15)
**AQ**	12.8 (5/39)	13.1 (5/38)*	10.5 (4/38)*	9.6 (3/31)	0 (0/30)
**SP**	10.2 (4/39)	44.4 (16/36)	33.3 (12/36)	17.1 (6/35)	6.1 (2/33)
**AQ+SP**	2.4 (2/83)	3.8 (3/80)*	6.3 (5/80)*	1.3 (1/75)^¶^	1.3 (1/75)
**AQ+AS**	3.6 (3/83)	2.5 (2/80)*	1.2 (1/79)*	1.5 (1/64)^¶^	0 (0/64)

CQ, Chloroquine, AQ, amodiaquine; SP, Sulphadoxine-Pyrimethamine; AS, artesunate; 95% CI, 95% confidence interval.

* On day 7 and day 14, significant difference to SP group (P < 0.05)

¶On day 21, significant difference to SP group (P < 0.05)

## Discussion

The main objective of this study was to compare, for the first time in Madagascar, the efficacy of two different combination therapies: AQ+SP, an inexpensive regimen that has proven to be efficacious in recent studies [13-16] and AQ+AS, an ACT regimen chosen by National Malaria Control Programme (NMCP) as first-line treatment for uncomplicated malaria. This clinical trial was designed to include follow-up for children in each treatment group enrolled within 28 days because it had been shown that antimalarial drug efficacy studies that limit follow-up to 14 days or less may significantly underestimate the risk of re-emergence [17].

The findings are the preliminary results of an extended study of the efficacy of antimalarial drugs for the treatment of uncomplicated falciparum malaria, which involves eight sites with differing levels of transmission intensity across Madagascar. The methodology used was based on the 2003 WHO protocol [11], with some modifications (compromise between the high transmission and the low to moderate transmission protocols): (i) children enrolled in the study were between 6 months and 15 years of age and (ii) of *P. falciparum *parasitaemia on inclusion was between 1,000 and 200,000/μl.

The first step was to update data concerning therapeutic efficacies of CQ, AQ and SP alone, because CQ is has long been widely used in Madagascar and recommended by the NMCP for the home management of presumed malaria in children under five years of age (HMM). Pre-packaged presentations of chloroquine are currently available for children from six to 11 months of age and for children from 12 to 59 months of age and either sold at an affordable price of US $0.025 (PaluStop^®^) or freely distributed at primary public health facilities (Ody Tazomoka^®^) [18]. SP has also been used, since 2005, for intermittent preventive treatment in pregnant women in all areas of Madagascar except the Central Highlands.

No severe side-effects attributable to study medication were found during the follow-up period in any of the treatment groups. CQ was significantly less effective than SP, AQ, AQ+SP and AQ+AS in treating uncomplicated falciparum malaria, with overall treatment failure of 35.9% within 14 days of follow up and 44.4% within 28 days (PCR-corrected). These data show a higher prevalence of chloroquine resistance than reported in previous studies [19-21] and a good effectiveness of SP and AQ, as had been observed in many areas in West Africa [22,23]. These results confirm the urgent need to replace pre-packaged CQ with another pre-packaged effective therapy. According to the NMCP of the Ministry of Health of Madagascar, this switch will be gradual with the introduction of pre-packaged artemisinin-based combination therapies (co-formulated presentation of the AQ+AS combination, COARSUCAM^©^, Sanofi-Synthélabo Groupe, Paris, France) from the beginning of 2008.

This study also confirms that the assumption that ACTs are always more effective than NACTs is not always true especially in settings, such as Madagascar, where SP or AQ monotherapies remain effective. It was observed that AQ+SP was as effective as AQ+AS, consistent with previous reports in Africa both in high transmission [14,15,24] and in low transmission areas [16]. No significant difference between the two combination treatments was observed in terms of efficacy, safety, tolerance, fever clearance, or haematological recovery. Moreover, no significant difference in gametocyte prevalence was found between the two combination treatments. The observed lower prevalence of gametocytes in AQ+SP and AQ+AS groups reflect the effect of the additional drug on gametocyte survival or development, as has been previously shown for AQ+AS [14] and AQ+SP [9,25].

Although AQ+AS produced faster parasite clearance than AQ+SP, the risk of new infection within the month after therapy was high with AQ+AS than AQ+SP treatment, even in a low transmission area such as Moramanga. This phenomenon has previously been observed in areas of high transmission [14,24]. According to Krishna [26] and Watkins [27], the prevention of new infections by AQ+SP is probably due to the long elimination half-lives of the two drugs, whereas with AQ+AS combination, AS is rapidly eliminated, leaving only AQ to provide post-treatment prophylaxis.

However, cost and availability of ACTs remain major concerns, and it appears that the sudden increase in demand for artemisinins may exacerbate these problems, at least in the short-term [7]. AQ+AS (0.51 US$) [28] is currently four times more expensive than AQ+SP (0.13 US$). In Madagascar, with a reported 2,114,400 cases of suspected malaria (2003), the use of AQ+SP instead of AQ+AS would reduce the annual antimalarial treatments costs by 800,000 US$. In view of the recent report by Tagbor and colleagues [29] there is hope that AQ+SP could serve as a safe and effective alternative for malaria treatment in pregnancy and for intermittent preventive treatment until the safety of ACTs for pregnant women has been established. Moreover, further studies should be carry out to assess the usefulness of AQ+SP pre-packed fixed-dose combination as alternative of SP alone in intermittent preventive treatment in infants and as alternative for CQ for the home management of presumed malaria in children under five years of age.

In conclusion, these results indicate that AQ+SP is as effective as AQ+AS, and thereby show that the inexpensive and widely available combination AQ+SP may still be appropriate for the treatment of uncomplicated malaria in Madagascar, an area where resistance to the drugs is relatively uncommon. This could have an important role in this country, where diagnostic services are not accurate and where much of the drugs administered go to patients who do not have malaria.

## Authors' contributions

Didier Ménard was involved in all stages of this study. Arsène Ratsimbasoa, Nohary Nina Harimanana Andrianina, Zakaherizo Ramiandrasoa, Arthur Randriamanantena and Noéline Rasoarilalao performed the field work. Martial Jahevitra performed molecular genotyping. Luciano Tuseo and Andrianirina Raveloson helped to compose the manuscript and gave constructive advice.
